# Binder-Free Fabrication of Prussian Blue Analogues Based Electrocatalyst for Enhanced Electrocatalytic Water Oxidation

**DOI:** 10.3390/molecules27196396

**Published:** 2022-09-27

**Authors:** Ruqia, Muhammad Adeel Asghar, Sana Ibadat, Saghir Abbas, Talha Nisar, Veit Wagner, Muhammad Zubair, Irfan Ullah, Saqib Ali, Ali Haider

**Affiliations:** 1 Department of Chemistry, Quaid-i-Azam University, Islamabad 45320, Pakistan; 2 Department of Biological Sciences, National University of Medical Sciences, Rawalpindi 46000, Pakistan; 3 Department of Physics and Earth Sciences, Jacobs University, 28759 Bremen, Germany; 4 Pakistan Academy of Sciences, 3-Constitution Avenue Sector G-5/2, Islamabad 44000, Pakistan

**Keywords:** electrocatalysis, water oxidation, oxygen evolution reaction, prussian blue analogues, surfactants, binder-free catalysts

## Abstract

Developing a cost-effective, efficient, and stable oxygen evolution reaction (OER) catalyst is of great importance for sustainable energy conversion and storage. In this study, we report a facile one-step fabrication of cationic surfactant-assisted Prussian blue analogues (PBAs) M_x_[Fe(CN)_5_CH_3_C_6_H_4_NH_2_]∙yC_19_H_34_NBr abbreviated as SF[Fe-Tol-M] (where SF = N-tridecyl-3-methylpyridinium bromide and M = Mn, Co and Ni) as efficient heterogeneous OER electrocatalysts. The electrocatalysts have been characterized by Fourier transform infrared (FT-IR) spectroscopy, powder X-ray diffraction (PXRD), scanning electron microscopy (SEM) coupled with energy dispersive X-ray (EDX) analysis, and X-ray photoelectron spectroscopy (XPS). In the presence of cationic surfactant (SF), PBAs-based electrodes showed enhanced redox current, high surface area and robust stability compared to the recently reported PBAs. SF[Fe-Tol-Co] hybrid catalyst shows superior electrochemical OER activity with a much lower over-potential (610 mV) to attain the current density of 10 mA cm^−2^ with the Tafel slope value of 103 mV·dec^−1^ than that for SF[Fe-Tol-Ni] and SF[Fe-Tol-Mn]. Moreover, the electrochemical impedance spectroscopy (EIS) unveiled that SF[Fe-Tol-Co] exhibits smaller charge transfer resistance, which results in a faster kinetics towards OER. Furthermore, SF[Fe-Tol-Co] offered excellent stability for continues oxygen production over extended reaction time. This work provides a surface assisted facile electrode fabrication approach for developing binder-free OER electrocatalysts for efficient water oxidation.

## 1. Introduction

The global energy demand is anticipated to be doubled due to the rapid expansion of technology and depletion of fossil fuels. To achieve “dual-carbon” goals, it is not only necessary to control carbon emissions from traditional fossil fuels, but also to find suitable alternative energy sources [1]. Hydrogen can be used as a promising clean energy source because of its high energy content and environmentally friendly nature, therefore, it has been extensively studied during the past decades. Incredible efforts have been devoted to the inexpensive and efficient production of hydrogen as well as to scrutinize some strategic techniques [2,3,4,5]. The oxygen evolution reaction (OER), a half-reaction of water electrolysis, is alleged to be the rate-limiting step that significantly impedes the overall efficiency of electrochemical hydrogen production [6]. High thermodynamic potential, as well as slow kinetics for OER leading to higher overpotential, necessitates the use of an efficient electrocatalyst to favor the reaction at lower overpotential values [7,8]. Furthermore, water electrolysis is reliant on pH and significant literature reports the use of basic and acidic conditions for oxygen evolution and hydrogen evolution reactions, respectively. However, extreme conditions of pH do not meet the green chemistry rules. So, the search for efficient and robust water oxidation catalysts (WOC) that could work under milder conditions is crucial for electrochemical energy devices [6,9]. IrO_2_ and RuO_2_ show excellent electrochemical performance, but their applications have been constrained due to their high cost and scarcity [10]. First-row transition metals such as manganese, cobalt, nickel, and iron-based materials, have drawn enormous considerations as appropriate electrocatalysts for the OER due to their low cost, high activity, and long-term stability [11,12,13]. Despite the high catalytic activities of metal oxides, particularly cobalt oxide [14], they have low stability and a high tendency to decompose in an acidic medium. Prussian blue (PB) and its analogues (PBAs, Na_2_M[Fe(CN)_6_], M = Fe, Co, Mn, Ni, Cu, etc.) belong to a large class of transition-metal hexacyanoferrates with an open framework structure, numerous redox-active sites, and strong structural stability. Therefore, due to their unique structural features, PB and its analogs have been thoroughly investigated as new alternative catalysts for energy-based applications [15].

Subsequently, the investigation of Prussian blue analogues (PBAs) as WOCs by the group of Galán-Mascarós is among the targeted studies showing that metal hexacyanometalates are robust and stable in a wide pH range [15]. However, PBAs present low current densities (~1 mA·cm^−2^ at overpotential >600 mV) due to their high crystallinity and low mechanical resistance attributing to their poor interfacial matching with the electrode surface. In follow-up studies by the group of Ferdi, pentacyanometallate-based PBA i.e., [CoFe(CN)_5_-PVP] exhibited a current density of 1 mA cm^−2^ at comparatively lower overpotential η = 510 mV [16]. The study further shows that the OER reaction occurs more efficiently on the surface of amorphous material than on that of a crystalline one, owing to more surface-active sites. This work provided the room to explore and investigate the effect of N-donor ligands on the water oxidation performance of PBAs. It has also been observed that conventional electrode fabrication using a binder, can lead to the undesirable electrode interface, i.e., higher resistance amid electrocatalyst and substrate, thereby lessening the active sites and flaking off the catalyst etc. Therefore, binder-free electrocatalysts are advantageous to boost the catalytic performances of PBAs [17,18]. Moreover, the in situ growth of a conductive layer i.e., graphene, CNTs and surfactants, etc., onto an electrode and electrocatalyst interface is an effective tactic to construct an electron pathway and regulate electron transference between them [17,18,19,20].

Surfactants can act as structure-directing agents and have a considerable influence on electrochemical reactions [21,22]. A surfactant-based conductive layer between the electrode and electrocatalysts has played a significant role in the enhanced film growth, increasing stability and with faster charge transfer kinetics [17].

Herein, we have investigated the effect of cationic surfactant i.e., N-tridecyl-3-methylpyridinium (SF) on the electrocatalytic performance of PBA films towards water oxidation reaction. We have developed the strategy of preparing binder-free, surfactant-assisted PBA films on the glassy carbon electrode (GCE). The addition of this surfactant immobilizes PBAs on the bare GCE and significantly enhances the charge transfer between the analyte and the electrode surface [23,24]. Thus, the fabrication of SF[Fe-Tol-M] films on GCE has shown significant improvement in electrochemical performance towards water oxidation in terms of currents density, redox activity, and electrochemical stability.

## 2. Experimental Section

### 2.1. Chemicals and Instruments

All the reagents and solvents (analytical grade) were purchased from Sigma-Aldrich (St. Louis, MO, USA) and used as received without any further purification. All salt solutions were prepared in deionized water (resistivity: 18 mΩ·cm) at 25 °C.

The electrochemical measurements were carried out on a Gamry 1010E potentiostat/galvanostat equipped with a standard three-electrode system comprising of platinum wire, Ag/AgCl (3 M KCl) and modified glassy carbon (2 mm diameter) as counter, reference, and working electrodes, respectively.

FT-IR absorption spectra of compounds were recorded on a Thermo Nicolet-6700 spectrophotometer in the frequency range of 4000–400 cm^−1^. The NMR spectra (^13^C and ^1^H) were recorded on a Bruker Advanced Digital 300 MHz spectrometer (Switzerland) using D_2_O as a solvent and the chemical shifts (δ) are given in ‘ppm’. Shimadzu-1800 double beam spectrophotometer was used to determine the characteristic peak positions in UV-visible region (200–800 nm). PXRD analyses were carried out on a PANalytical X’Pert multipurpose X-ray diffraction system, in the 2θ range of 10–80°. SEM images were obtained by using FEI Nova SEM-230 coupled with Bruker EDX system at an accelerating voltage of 3 kV. XPS analyses of the synthesized PBAs were performed by mounting the samples onto a holder with a conducting carbon tape to avoid surface charging during the measurements. The samples were dispersed in acetone and drop-casted onto a silicon wafer. The solvent was dried under nitrogen flow. In order to prevent charging during the photoelectron measurement, a highly doped silicon wafer with natural oxide was used as a substrate. After preparation of the sample, it was introduced to the UHV chamber. The UHV chamber operating at a vacuum pressure of ~1 × 10^−9^ mbar was equipped with a water-cooled X-ray gun (Mg/Al, Specs XR 50) and a hemispherical analyzer (Specs Phoebos 100). For excitation, the MgKα radiation (1253.6 eV) source was used in this experiment. Large lens mode was used for the detection of the photoelectron with pass energy of 30 eV. For data analysis the CASA-XPSTM software was used. The measured data was fitted using the CASA XPSTM software and the background was subtracted using Shirley’s methods. A simplified Voigt function was used for fitting with sample width at half maximum (FWHM) for doublet and the ratio between 2p_3/2_ and 2p_1/2_ was found to be 0.5. Electrocatalytic performance of PBA-based modified electrodes was tested on Gamry interface 1010E on the workstation at ambient temperature.

### 2.2. Synthesis of the Surfactant

Pyridine-based cationic surfactant was synthesized (Scheme S1) according to our reported method with slight modification [25,26]. Briefly, surfactant was synthesized by refluxing equimolar amounts of 3-methyl pyridine (0.20 g; 2.15 mmol) and tridecyl bromide (0.57 g; 2.15 mmol) in 25 mL dried toluene as a solvent for 10 h. After cooling to room temperature, the reaction mixture was filtered off and the brownish viscous compound was obtained by rotary evaporation. The purity of SF was confirmed by FT-IR and NMR spectroscopy (see Supplementary Information).

### 2.3. Synthesis of the Sodium Pentacyanoammineferrate(II)

Sodium pentacyanoammineferrate(II) (Na_3_[Fe(CN)_5_NH_3_]·3H_2_O) abbreviated as [Fe-NH_3_] as a precursor was synthesized from sodium pentacyanonitrosylferrate(III) dihydrate (Na_2_[Fe^III^(CN)_5_NO]·2H_2_O) abbreviated as [Fe-NO] according to the reported procedure with slight modification [16]. Briefly, 15 g of [Fe-NO] was dissolved in 40 mL of water followed by the addition of 6 g of NaOH under constant stirring at 10 °C. Afterwards, NH_4_OH (25% *v/v*) was added to this solution until saturation, followed by the addition of cold methanol [16]. The resulting yellow precipitates were aged overnight at 0 °C and recrystallized using NH_4_OH/CH_3_OH solution that were finally dried under vacuum (Scheme S2). The obtained yield was calculated to be 50%.

### 2.4. Synthesis of Sodium Pentacyano(methylaniline)ferrate (II) [Fe-Tol]

The Prussian blue precursor was synthesized by a ligand substitution reaction of [Fe-NH_3_] with 2-methylaniline by slight modification in a reported procedure [16,27,28]. The 2-methylanaline (0.65 mL; 6.14 mmol) was added slowly into the [Fe-NH_3_] solution (2 g; 6.14 mmol) under constant stirring at room temperature (Scheme S3). The reaction was allowed to continue in a covered flask for 24 h. The reaction mixture containing the green-colored precipitates was then concentrated to a 5 mL solution and was washed with cold methanol followed by centrifugation (6000 rpm) to remove unreacted [Fe-NH_3_]. Then, cold diethyl ether (250 mL) was added to the suspension (approximately 50 mL) under constant stirring. The precipitates were isolated and dried under vacuum overnight at room temperature which resulted in a green-colored powder with a yield of about 63%.

### 2.5. Modification of Electrode for OER

The deposition of the PBA films onto the GCE was accomplished by first placing a drop of surfactant solution (in methanol) followed by the addition of 10 µL of 10 mM [Fe-Tol] and 15 mM of desired metal salt solutions i.e., Co(NO_3_)_2_·6H_2_O, Ni(NO_3_)_2_·6H_2_O and Mn(NO_3_)_2_·4H_2_O to prepare their corresponding SF[Fe-Tol-Co], SF[Fe-Tol-Ni] and SF[Fe-Tol-Mn] films, respectively. The adsorption of surfactant to GCE occurs through the attachment of polar head groups of the surfactant to the hydrophilic ionic groups on GCE, which provides hemimicelles of the surfactant. During hemimicelles’ formation, the head groups expose themselves to the aqueous solution because of interactions between the hydrocarbon chains [29]. Cyclic voltammetry (CV) measurements were recorded in 50 mM phosphate buffer (pH 7) containing 1 M NaNO_3_ as electrolyte between 0–1.5 V versus Ag/AgCl at the scan rate of 50 mVs^−1^.

## 3. Results and Discussion

### 3.1. Characterization

**Figure 1:**
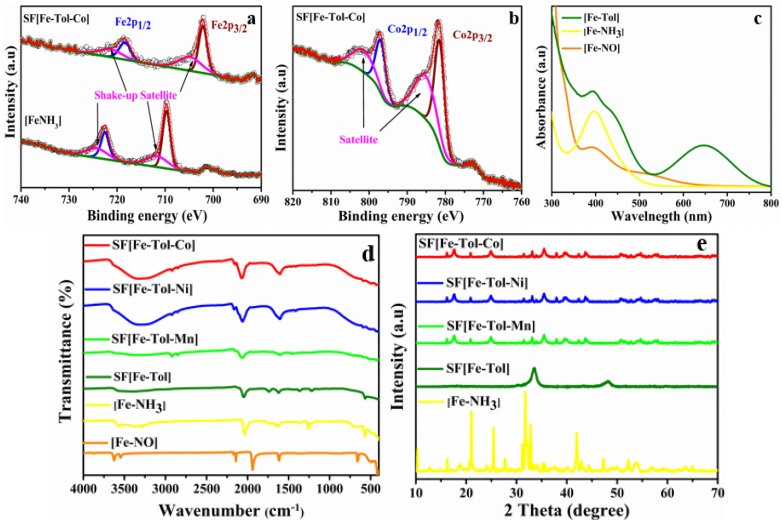
(**a**) XPS spectra of [Fe-NH_3_] and SF[Fe-Tol-Co] for Fe, (**b**) XPS spectrum of SF[Fe-Tol-Co] for Co, (**c**) overlaid UV-visible spectra of [Fe-NO], [Fe-NH_3_] and [Fe-Tol], (**d**) overlaid FT-IR spectra of [Fe-NO], [Fe-NH_3_], [Fe-Tol] and SF[Fe-Tol-M] and (**e**) XRD patterns for [Fe-NH_3_], [Fe-Tol] and SF[Fe-Tol-M] (where M = Mn, Co, Ni).

The surfactant-assisted PBAs were synthesized in different steps and characterized by several techniques. Firstly, [Fe-NO] was reduced to [Fe-NH_3_] (Scheme S1) and then -NH_3_ ligand was substituted by 2-methylaniline (Scheme S2). The reduction of [Fe-NO] and oxidation state of metals in SF[Fe-Tol-M] have been confirmed by XPS. In the XPS spectrum of [Fe-NH_3_], a doublet with the binding energy of 722.9 and 709.8 eV for the Fe 2p_1/2_ and 2p_3/2_, respectively, with corresponding shake-up satellites are attributed to the Fe^2+^ state validating the complete reduction of Fe^3+^ in [Fe-NO] to [Fe-NH_3_] (Figure 1a). Furthermore, the spectrum of SF[Fe-Tol-Co] mainly exhibits a doublet with a binding energy of 722.8 and 709.7 eV for Fe 2p_1/2_ and 2p_3/2_, which is attributed to the 2+ oxidation state of iron in SF[Fe-Tol-Co] and confirms that the Fe^2+^ is retained after PBA formation [30]. Similarly, the spectrum of SF[Fe-Tol-Co] is shown in Figure 1b which exhibits a doublet for Co with a binding energy of 797.1 and 781.4 eV for Co 2p_1/2_ and 2p_3/2_, respectively corresponding to the 2+ oxidation state of cobalt [31,32]. In addition to the main photoelectron peak, shake-up satellite peaks are also visible at the higher binding energies in the spectrum. The XPS overview spectra of SF[Fe-Tol-M] (M = Co, Ni and Mn) are shown in Figure S2, where corresponding photoelectron peaks and Auger peaks are indicated. The substitution of 2-methylaniline moiety has further been confirmed by UV-visible and FT-IR spectroscopy. In the UV-visible spectra (Figure 1c), broad absorption at 645 nm, corresponding to the n→π* transition for [Fe-Tol] shows the presence of 2-methylaniline in [Fe-Tol] complex [33]. Similarly, the band observed at 395 nm is accredited to the metal to ligand charge transfer (MLCT) transition i.e., ${\mathrm{Fe}}^{\mathrm{II}}\to \pi *$ (L), which is typical for the coordination of Fe^II^(CN)_5_ to organic ligand [34] that confirms the successful replacement of -NH_3_ by 2-methylanilie. In the FT-IR spectrum of [Fe-Tol] (Figure 1d), the mild shift of $v({\mathrm{CN}}^{-}$) to a higher frequency compared to that of [Fe-NO] and [Fe-NH_3_] and the appearance of *v*(CH) at 2968 cm^−1^ further supports the substitution of the 2-methylaniline group. Moreover, perceptible bands observed in the range of 1600–1360 cm^−1^ for aromatic ring and CH bending endorse the replacement of the NH_3_ group [35]. In case of SF[Fe-Tol-M], the $v({\mathrm{CN}}^{-}$) has been shifted to 2067–2063 cm^−1^ due to the interaction of Fe^2+^ − CN − M^2+^ [36]. The broadband in the range of 3400–3200 cm^−1^ and 1610–1595 cm^−1^ are attributed to stretching and bending modes of the -OH group of water molecules present in the interstitial spaces of SF[Fe-Tol-M].

PXRD analysis was performed to understand the morphological features of the synthesized materials. The PXRD patterns of the as-synthesized PBAs and their lattice structures are given in Figure 1e. All PBAs demonstrated identical XRD patterns, identified as a face-centered cubic lattice. The crystallinity of the synthesized PBAs is lower than that of precursors and hexacyanoferrates, which might be attributed to the presence of organic ligand resulting in lower crystallinity [37].

**Figure 2:**
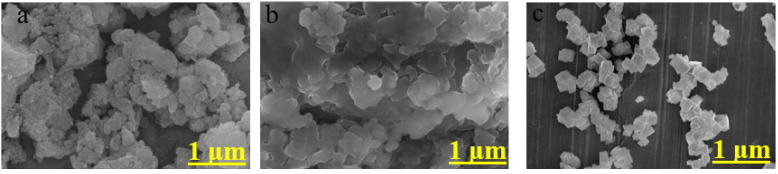
SEM image of the (**a**) SF[Fe-Tol-Co], (**b**) SF[Fe-Tol-Ni], (**c**) SF[Fe-Tol-Mn].

The SEM images of PBAs shown in Figure 2 reveal the significant clustering in all SF[Fe-Tol-M] samples. The clustering is more in SF[Fe-Tol-Ni] than in SF[Fe-Tol-Co] and SF[Fe-Tol-Mn] which could be due to the more agglomeration upon drying of spheres in it. Further, these images suggest sheets/cubes-like structures. The EDX analysis has further been performed to determine the elemental composition of the SF[Fe-Tol-M] given in Table S1. During the EDX measurement, different areas were focused to identify the corresponding metal (M) peaks as can be seen in the EDX spectra shown in Figure S3. Considering the percentage content of Na, Mn, Co, Ni and Fe for each catalyst, the molecular formulae are approximated as ${\mathrm{Co}}_{1.28}\left[\mathrm{Fe}{\left(\mathrm{CN}\right)}_{5}\mathrm{CH}₃C₆H₄\mathrm{NH}₂\right]\cdot 0.44C_{19}H_{34}\mathrm{NBr}$, ${\mathrm{Ni}}_{1.26}\left[\mathrm{Fe}{\left(\mathrm{CN}\right)}_{5}\mathrm{CH}₃C₆H₄\mathrm{NH}₂\right]\cdot 0.46C_{19}H_{34}\mathrm{NBr}$ and ${\mathrm{Mn}}_{0.88}\left[\mathrm{Fe}{\left(\mathrm{CN}\right)}_{5}\mathrm{CH}₃C₆H₄\mathrm{NH}₂\right]\cdot 1.2C_{19}H_{34}\mathrm{NBr}$ [38].

### 3.2. Electrocatalytic Performance

**Figure 3:**
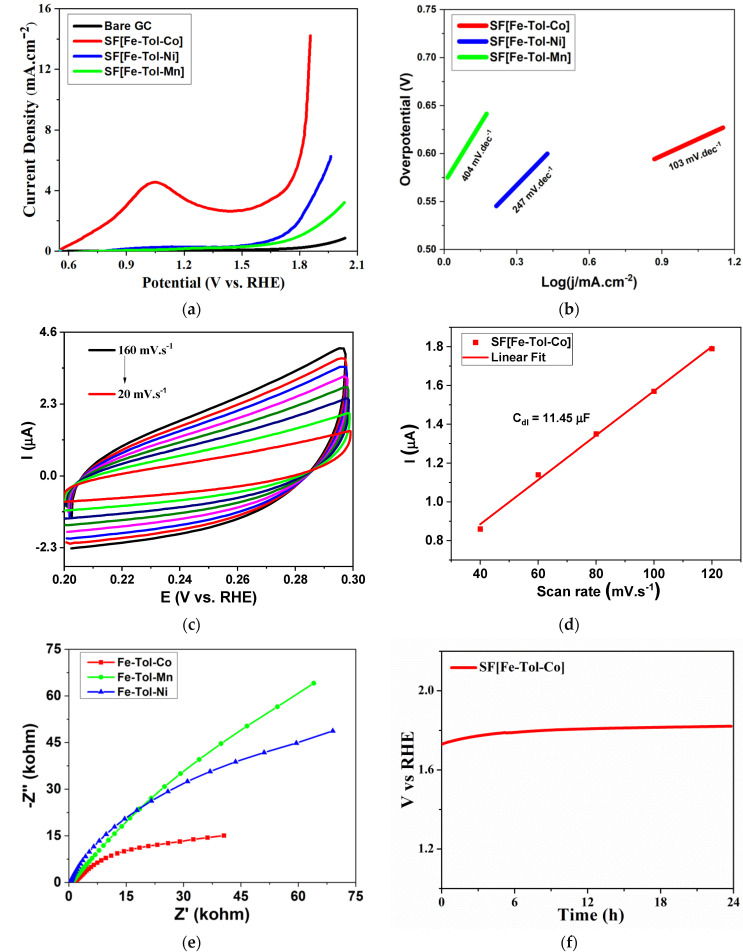
(**a**) Linear sweep voltammograms of bare GCE and SF[Fe-Tol-M], (**b**) Tafel plots of SF[Fe-Tol-M], (**c**) cyclic voltammograms of SF[Fe-Tol-Co] in the non-faradaic potential region at the scan rates ranging from 20 to 160 mV·s^−1^, (**d**) charging currents measured at the potentials 0.25 V vs. Ag/AgCl plotted as a function of scan rates for SF[Fe-Tol-Co], (**e**) Nyquist plots of SF[Fe-Tol-M] in the frequency range from 100 mHz to 100 kHz and (**f**) chronopotentiometric measurement for SF[Fe-Tol-Co] performed to generate constant current density of 5 mV·cm^−2^ for 24 h.

The transition metal-based (especially the ones based on iron, cobalt, and nickel etc.) OER electrocatalysts undergo a pre-oxidation of the low-valence metal before the OER catalysis and form the catalytically active high-valence species. The high-valence active species formed in situ accumulates and hence leads to the substantial increment in the OER activity [39,40]. The group of Galán Mascarós put forward a phase conversion approach to transform Co(OH)_1.0_(CO_3_)_0.5_·nH_2_O into CoFe PBA thin film, resulting in an improved OER activity [41]. Up untill now, along with the rapid and scalable synthesis approach of the PBAs-based catalysts, related elucidation of the pre-oxidation process before OER catalysis is also needed [39]. We have fabricated PBAs using cationic surfactant with tunable compositions. Linear sweep voltammograms of SF[Fe-Tol-Co], SF[Fe-Tol-Ni] and SF[Fe-Tol-Mn] are shown in Figure 3a. It can be seen from the figure that the current density of 10 mA·cm^−2^ is achieved with an overpotential of 610 mV vs. RHE for SF[Fe-Tol-Co], which is superior to the nickel- and manganese-based analogues. However, SF[Fe-Tol-Ni] and SF[Fe-Tol-Mn] exhibited current density of 2.91 and 1.26 mA·cm^−2^, respectively, at the same overpotential value. Interestingly, SF[Fe-Tol-Co] delivered the maximum current density of 14.23 mA·cm^−2^ that even outperformed the other reported PBAs (see Table S2). The favorable reaction kinetics during OER catalysis and superior activity of SF[Fe-Tol-Co], compared to other two PBAs, is further supported by the Tafel slopes values (Figure 2b), which are calculated to be 103, 247, and 404 mV·dec^−1^ for SF[Fe-Tol-Co], SF[Fe-Tol-Ni] and SF[Fe-Tol-Mn], respectively, where cobalt-based electrocatalyst presented the lowest value of Tafel slope.

Furthermore, the electrochemically active surface area (ECSA) considerably affects the electrocatalytic activity of the electrode. Higher ECSA leads to more active sites for possible electrochemical processes and vice versa. Therefore, ECSA has been calculated for SF[Fe-Tol-M] by performing CV experiments in the non-faradaic region from 0.2 to 0.3 V versus RHE at scan rate ranging from 20 to 160 mVs^1^ as shown in Figure 3c and Figure S4a,c. The slopes of current (mA) versus scan rate (mVs^−1^) plot at a potential of 0.25 V vs. RHE, provided double-layer capacitance (C_dl_), as shown in Figure 3d and Figure S4b,d for SF[Fe-Tol-Co], SF[Fe-Tol-Ni] and SF[Fe-Tol-Mn], respectively. The ECSA and roughness factor (RF) is determined by using Equations (S1) and (S2) [42,43]. SF[Fe-Tol-Co] displayed the highest ECSA and RF followed by SF[Fe-Tol-Ni] and SF[Fe-Tol-Mn], respectively. Table 1 presents the C_dl_, ECSA, and RF, for all the prepared electrodes [42,44].

The high current density, significantly lower over-potential value, and high ECSA of SF[Fe-Tol-Co] compared to the recently reported ones, can be attributed to the binder-free fabrication approach. A conductive layer of the surfactant also favors the charge transfer and hence reduces the free energy barrier [22]. A comparison of the catalytic activity of the synthesized PBAs with the reported PBAs is presented in Table S2.

The study of the reaction kinetics is crucial to understand the OER activity, which is investigated by the electrochemical impedance spectroscopy (EIS). The EIS analyses for SF[Fe-Tol-M] modified electrodes have been performed in phosphate buffer at pH 7 to obtain the Nyquist plot at in the frequency range from 100 mHz to 100 kHz providing a small AC signal of 5 mV (Figure 3e). The plot demonstrated that the charge transfer resistance (R_ct_) value is the lowest for SF[Fe-Tol-Co] as depicted by its smallest semicircle in the high-frequency region. The trend observed for the R_ct_ values is found to be SF[Fe-Tol-Co] > SF[Fe-Tol-Ni] > SF[Fe-Tol-Mn]. These results distinctly signified that the intermediate species formation and the overall OER rate are the most efficient for SF[Fe-Tol-Co], followed by SF[Fe-Tol-Ni], and SF[Fe-Tol-Mn], respectively [43]. Finally, to evaluate the long-term stability, chronopotentiometric measurement was carried out for SF[Fe-Tol-Co] under the steady generation of oxygen, as given in Figure 3f. The stability test was performed by applying constant current corresponding to j value of 5 mA·cm^−2^ and the corresponding potential was measured. The electrocatalyst showed good stability in the galvanostatic measurements for 24 h during continuous OER.

## 4. Conclusions

Many non-oxo bridged transition metal coordination networks of hexa- and pentacyanometalates are well known for their electrocatalytic OER performances. In this work, the surfactant-assisted PBAs of earth-abundant transition metals as heterogeneous electrocatalysts have been synthesized by exploiting a binder-free approach. This strategy employed cationic surfactant to facilitate the modification of GCE by PBAs film that subsequently boosts their electrochemical properties towards OER electrocatalysis, as compared to the conventional PBAs. The catalytic current density of 10 mA·cm^−2^ was achieved at much lower overpotential i.e., 610 mV for SF[Fe-Tol-Co], while 1 mA cm^−2^ was achieved at 480 mV, 570 mV for SF[Fe-Tol-Ni] and SF[Fe-Tol-Mn] modified electrodes, respectively. The high current density, significantly lower over-potential value, and high ECSA of SF[Fe-Tol-Co] compared to the recently reported PBAs, can be attributed to the binder-free fabrication approach. The fabrication procedure reported herein unveils a facile route for designing advanced binder-free electrocatalysts with easily available transition metals with tuned performance. Further, the strategy will be used to introduce a series of robust and efficient catalysts to the field of water oxidation and can be extended to other materials and substrates as well.

## Figures and Tables

**Table 1:** C_dl_, ECSA, and RF values for SF[Fe-Tol-M].

Sample Code	Capacitance (C_dl_) (µF)	ECSA (cm^2^)	Roughness Factor (RF)
SF[Fe-Tol-Co]	11.65	0.58	19.33
SF[Fe-Tol-Ni]	10.10	0.50	16.67
SF[Fe-Tol-Mn]	4.50	0.22	7.33

## Supplementary Materials

The following supporting information can be downloaded at: https://www.mdpi.com/article/10.3390/molecules27196396/s1, Scheme S1: Synthesis of N-tridecyl-3-methylpyridiniumbromide (SF); Scheme S2: Synthesis of Na_3_[Fe(CN)_5_NH_3_]·3H_2_O from Na_2_[Fe(CN)_5_NO]·2H_2_O; Scheme S3: Synthesis of [Fe-Tol] using [Fe-NH_3_] as a precursor; Scheme S4: Synthesis of PBAs; Figure S1: (a) ^1^H-NMR and (b) ^13^C-NMR of SF; Figure S2: Overview of XPS spectra of the M 2p region for [Fe-Tol-M] (where M = Mn, Co, Ni); Figure S3: EDX analysis of (a) SF[Fe-Tol-Co] (b) SF[Fe-Tol-Ni] and (c) SF[Fe-Tol-Mn]; Figure S4: (a,c) Cyclic voltammograms of SF[Fe-Tol-Ni] (a) and SF[Fe-Tol-Mn] (c) in the non-faradaic potential region at scan rates 20–200 mVs^−1^. (b,d) Charging current plotted as a function of scan rates for SF[Fe-Tol-Ni] and SF[Fe-Tol-Mn] modified electrode recorded in 50 mM phosphate buffer (pH 7); Table S1: Elemental composition of SF[Fe-Tol-M] by EDX measurement; Table S2: Comparison of the catalytic parameters of F[Fe-Tol-Co] with some other water oxidation electrocatalysts. References [45,46,47] are cited in Supplementary Materials.
