# Supplementary material for: Binder-Free Fabrication of Prussian Blue Analogues Based Electrocatalyst for Enhanced Electrocatalytic Water Oxidation
Source: Molecules. 2022 Sep 27;27(19):6396. doi: 10.3390/molecules27196396 (PMC9571080; doi:10.3390/molecules27196396)
Supplement: Supplementary file 1 [file molecules-27-06396-s001.zip › molecules-1904219-supplementary.pdf]

Binder-free Fabrication of Prussian Blue Analogues-based Electrocatalyst for Enhanced Electrocatalytic Water Oxidation

Ruqia,[1,#] Muhammad Adeel Asghar,[1,#] Sana Ibadat,[1] Saghir Abbas,[2] Talha Nisar,[3] Viet Wagner,[3] Muhammad Zubair,[1] Irfan Ullah,[1] Saqib Ali,[1,4*] Ali Haider[1,4*]

[1]Department of Chemistry, Quaid-i-Azam University, Islamabad, 45320, Pakistan.

[2]Department of Biological Sciences, National University of Medical Sciences, 46000, Rawalpindi, Pakistan.

[3]Physics and Earth Sciences, Jacobs University Bremen, Campus Ring 1, 28759 Bremen, Germany.

[4]Pakistan Academy of Science, 3-Constitution Avenue Sector G-5/2, Islamabad 44000, Pakistan

[#] Both contributed equally to the work

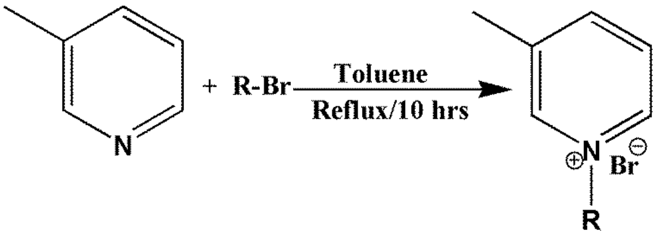

Where R = $-C_{13}H_{27}$

Scheme S1. Synthesis of N-tridecyl-3-methylpyridiniumbromide (SF).

Spectroscopic data of SF.

FT-IR (v (cm^{-1})): 1161 (C-N stretching), 1637 (C=N stretching), 728 (long alky-chains).

FT-NMR: ^{1}H-NMR (300 MHz, $CDCl_3$, δ-ppm); 9.34 (1H, H1, s), 8.22 (1H, H3, d), 3J[H1, H1] = 8.2Hz, 7.99 (2H, H4, t), 3J[H1, H1] = 7.97Hz, 9.16 (1H, H5, d), 3J[H1, H1] = 9.15 Hz, 3.3 (3H, H6, s), 4.81(2H, H7, t) = 4.78 Hz, 3.3-1.10 (22H, H8-18, m), 0.76 (3H, H19, t), 3J[1H, 1H] = 0.74 ^{13}C-NMR (77.5 MHz, $CDCl_3$, δ-ppm); 145.6 (C1), 139.5 (C2), 142.2 (C3), 127.9 (C4), 144.3 (C5), 25.8 (C6), 61.3 (C7), 34.1-18.6 (C8)

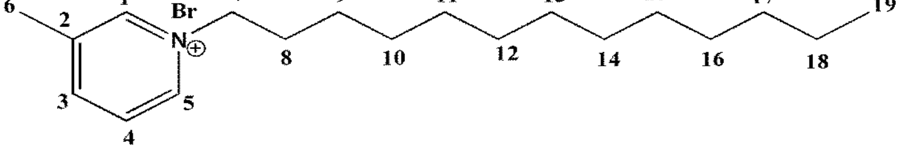

Structure of synthesized SF with the numbering of carbon/proton atoms for NMR spectral interpretation.

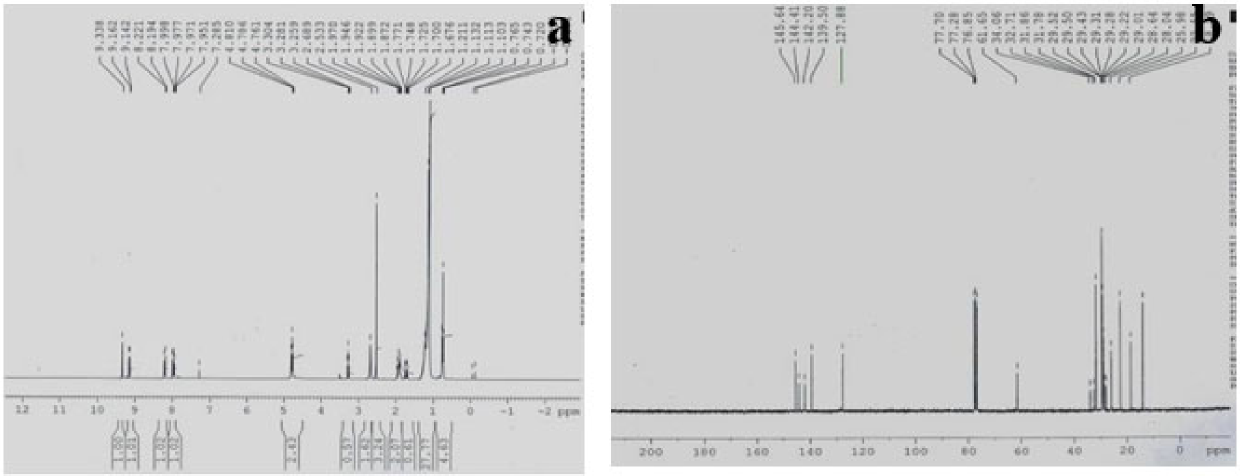

Figure S1. (a) ^{1}H-NMR and (b) ^{13}C-NMR of SF.

$$\mathbf{Na_2[Fe(CN)_5NO].2H_2O + 2NH_3 + NaOH \longrightarrow Na_3[Fe(CN)_5NH_3].3H_2O + 2N_2 + 4H_2O}$$

Scheme S2. Synthesis of $Na_3[Fe(CN)_5NH_3].3H_2O$ from $Na_2[Fe(CN)_5NO].2H_2O$.

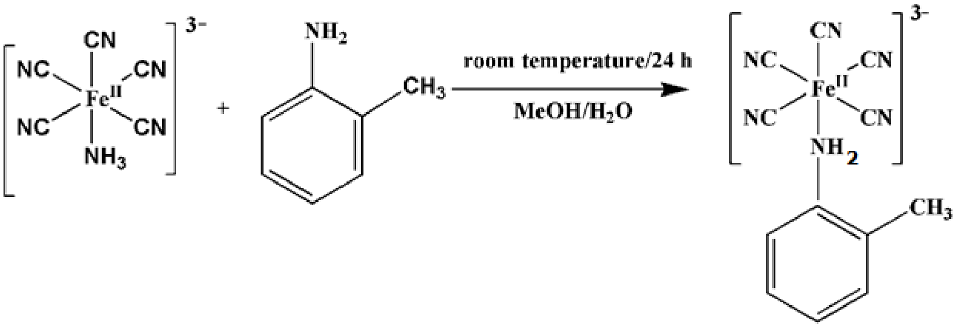

Scheme S3. Synthesis of [Fe-Tol] using $[Fe\text{-}NH_3]$ as a precursor.

$$\mathbf{Fe(CN)_5NC_7H_9 + MCl_2.xH_2O + C_{19}H_{34}N^+Br^- \xrightarrow{\text{Room temperature}} (M)_x(C_{19}H_{34}N)_y[Fe(CN)_5NC_7H_9]_z\ .nH_2O}$$

M= Mn, Co and Ni

Scheme S4. Synthesis of PBAs.

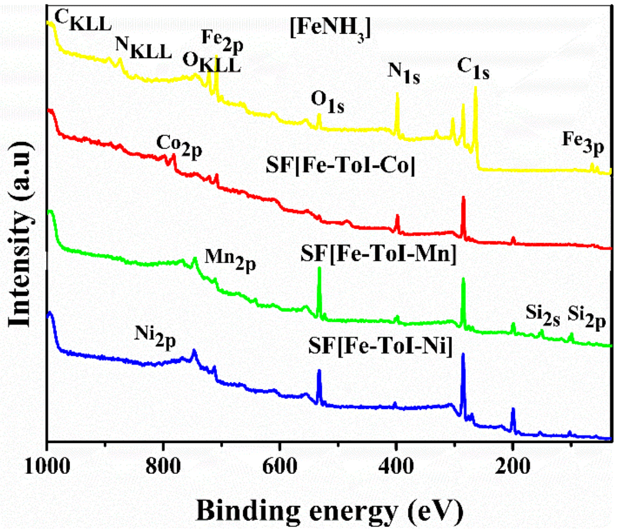

Figure S2. Overview of XPS spectra of the M 2p region for [Fe-Tol-M] (where M = Mn, Co, Ni).

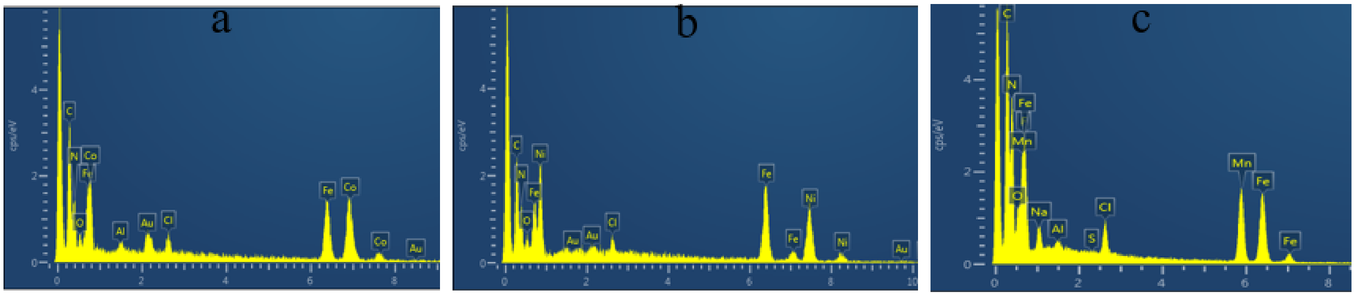

Figure S3. EDX analysis of (a) SF[Fe-Tol-Co] (b) SF[Fe-Tol-Ni] and (c) SF[Fe-Tol-Mn].

Table S1. Elemental composition of SF[Fe-Tol-M] by EDX measurement.

Name	Element	Apparent concentration	% Weight
SF[Fe-Tol-Co]	C	8.29	26.84
	N	21.33	17.38
	Fe	16.2	19.60
	Co	21.43	26.59
SF[Fe-Tol-Ni]	C	6.37	23.83
	N	20.1	16.25
	Fe	19.86	22.90
	Ni	24.45	30.72
SF[Fe-Tol-Mn]	C	3.40	26.13
	N	12.31	30.37
	Fe	3.44	15.71
	Mn	1.99	9.19

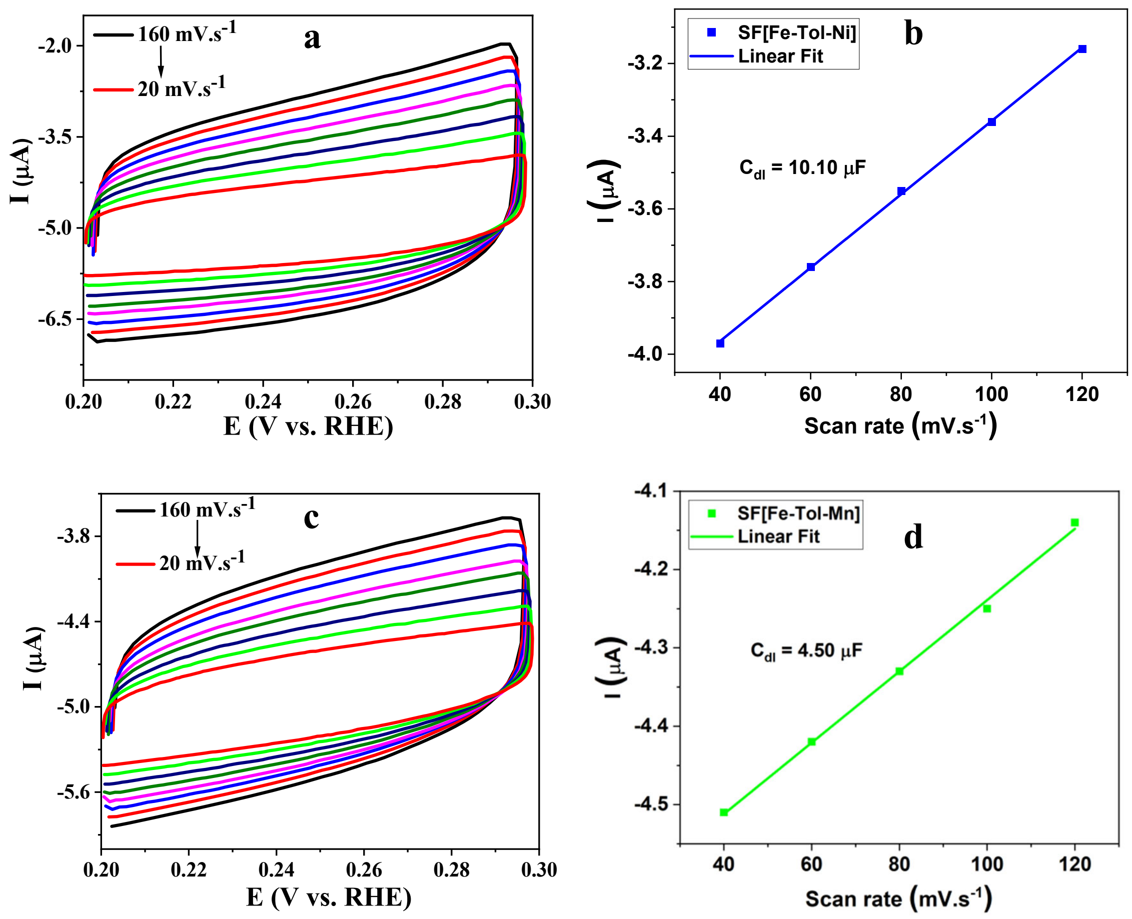

Figure S4. (a, c) Cyclic voltammograms of SF[Fe-Tol-Ni] (a) and SF[Fe-Tol-Mn] (c) in the non-faradaic potential region at scan rates 20-200 mV.s^{-1}. (b, d) Charging current plotted as a function of scan rates for SF[Fe-Tol-Ni] and SF[Fe-Tol-Mn] modified electrode recorded in 50 mM phosphate buffer (pH 7).

Determination of ECSA and roughness factor:

$$\mathrm{ECSA} = \frac{C_{dl}}{C_s} \qquad \text{S1}$$

$$\text{Roughness factor} = \frac{\mathrm{ECSA}}{A} \qquad \text{S2}$$

Where C_s is specific surface area and its value 20 μF.cm^{-2} is taken from the literature [1].

Table S2. Comparison of the catalytic parameters of F[Fe-Tol-Co] with some other water oxidation electrocatalysts [2]

Catalyst	Formula	Onset Potential (mV)	Overpotential (mV)@ 1 $mA.cm^{-2}$	pH	Tafel Slope ($mV.dec^{-1}$)	Preparation method	Ref.
SF[Fe-Tol-Co]	$Co_{1.32}[Fe-(CN)_5Tol].0.18SF$	325	610@10	7	103	Drop-cast	Present work
[Co-Fe]	$K_{2x}Co_{2-x}[Fe(CN)_6]$ (0.85 < x < 0.95)	N/A	>600	7	85-95	Electrodeposition	[3]
[Co-Fe(CN)5-P4VP]	$Co_{1.5}[Fe(CN)_5P4VP]$	360	510	7	111	Precipitation + Spin-coating	[4]
[Co-Fe]	$Co_{1.5}[Fe(CN)_6]$	N/A	500	7	91	Chemical etching of $Co(OH)(CO_3)_{0.5}$	[5]
[Co-Co^{III}]	$K_{0.38}Co_{1.31}[Co(CN)_6]$	283	565	7	99	Precipitation + drop-cast	[6]
[Co-Cr^{III}]	$K_{0.41}Co_{1.29}[Cr^{III}(CN)_6]$	303	598	7	96	Precipitation + drop-cast	[6]
[Co-Fe^{III}]	$K_{0.31}Co_{1.35}[Fe^{III}(CN)_6]$	323	717	7	127	Precipitation + drop-cast	[6]
[Co-Fe^{II}]	$K_{0.70}Co_{1.65}[Fe^{II}(CN)_6]$	343	1079	7	121	Precipitation + drop-cast	[6]

References

1.P. Connor, J. Schuch, B. Kaiser, W. Jaegermann. The determination of electrochemical active surface area and specific capacity revisited for the system MnO_x as an oxygen evolution catalyst. *Z. fur Phys. Chem*. 2020, 234(5), 979-94.

2. T. G. Ghobadi, E. Ozbay, F. Karadas. How to Build Prussian Blue-Based Water Oxidation Catalytic Assemblies? Common Trends and Strategies. *Eur J. Chem.* 2021, 27(11), 3638-49.

3. S. Pintado, S. F. Goberna , E.C. Adán, G. Mascarós. Fast and persistent electrocatalytic water oxidation by Co–Fe Prussian blue coordination polymers. *J. Am. Chem. Soc.* 2013, 135(36), 13270-73.

4. M. Aksoy, S.V.K. Nune, F. Karadas. A novel synthetic route for the preparation of an amorphous Co/Fe prussian blue coordination compound with high electrocatalytic water oxidation activity. *Inorg. Chem*. 2016, 55(9), 4301-07.

5. L. Han, P. Tang, A. Carmona, B. R. García, M. Torréns, R. Morante, G. Mascarós. Enhanced activity and acid pH stability of Prussian blue-type oxygen evolution electrocatalysts processed by chemical etching. *J. Am. Chem. Soc.* 2016, 138(49), 16037-45.
6. E.P. Alsaç, E. Ülker, S.V. Nune, Y. Dede, F. Karadas. Tuning the electronic properties of prussian blue analogues for efficient water oxidation electrocatalysis: experimental and computational studies. *Eur. J. Chem.* 2018, 24(19), 4856-63.
